# Maternal Knowledge, Attitudes, and Practices Towards the Prevention of Birth Defects in Eastern Cape, South Africa: A Multi-Level Contextual Analysis

**DOI:** 10.3390/ijerph23060742

**Published:** 2026-06-01

**Authors:** Thando Tetana, Muambangu Jean Paul Milambo, Longo-Mbenza Benjamin

**Affiliations:** Department of Public Health, Faculty of Medicine and Health Sciences, Walter Sisulu University, Mthatha 5100, Eastern Cape, South Africa

**Keywords:** birth defects, mixed methods, maternal knowledge, antenatal care, traditional beliefs, rural health, South Africa, public health, maternal health, risk factors

## Abstract

**Highlights:**

**Public health relevance—How does this work relate to a public health issue?**

- Birth defects remain a significant but under-recognized public health challenge in rural South Africa, particularly in low-resource settings.
- Maternal knowledge, attitudes, and practices (KAP) play a critical role in the prevention and early management of birth defects.

**Public health significance—Why is this work of significance to public health?**

- The study identifies major gaps in awareness of modifiable risk factors such as smoking, medication use, and advanced maternal age.
- Cultural beliefs and widespread reliance on traditional healers strongly influence maternal health behaviors and perceptions of birth defects.

**Public health implications—What are the key implications or messages for practitioners, policy makers, and/or researchers in public health?**

- There is a need for culturally sensitive health education interventions to improve maternal knowledge and preventive practices.
- Strengthening antenatal care services and integrating traditional healers into public health strategies may enhance prevention efforts and health outcomes.

**Abstract:**

**Background:** Birth defects remain a major global public health concern, particularly in low-resource settings where awareness and preventive practices are limited. Maternal knowledge, attitudes, and practices (KAP) are critical in the prevention and management of birth defects. This study explored contextual factors influencing maternal KAP using a mixed-methods approach in three rural districts of the Eastern Cape, South Africa. **Methods:** A convergent mixed-methods cross-sectional study was conducted among 72 mothers selected through purposive sampling. Quantitative data were collected using a structured questionnaire administered in English only, covering socio-demographic characteristics, obstetric history, knowledge, and preventive practices. Qualitative data were obtained through interviews exploring beliefs, perceptions, and cultural explanations of birth defects. Quantitative data were analysed using descriptive statistics and linear regression analysis to identify factors associated with birth defects, while qualitative data were thematically analysed to provide contextual understanding. **Results:** Most participants resided in the Amathole district (63.89%), followed by Alfred Nzo (18.06%) and Joe Gqabi (18.06%). Most women were aged between 20 and 35 years (52.78%), while 15.28% were younger than 20 years and 6.94% were older than 45 years. Over half of the respondents were single (55.56%), 34.72% were married, and the remainder were either separated (4.17%) or divorced (5.56%). Numerous participants had primary education (56; 77.78%), followed by secondary (11; 15.28%) and tertiary education (5; 6.94%). The majority were unemployed (56; 77.78%), while smaller proportions were employed (10; 13.89%) or engaged in other income-generating activities (6; 8.33%), indicating limited participation in formal employment among respondents. Nearly all participants (95.83%) had experienced pregnancy, with 70.83% reporting pregnancy-related complications. Only 2.78% reported having a child with a birth defect, while 90.28% reported a family history of birth defects. Knowledge of genetic causes was relatively high (69.23%), but awareness of modifiable risk factors was limited. Although 93.06% recognized alcohol use during pregnancy as harmful, fewer participants identified smoking or medication use (18.06%) and advanced maternal age (26.39%) as risk factors. Only 13.89% acknowledged the preventive role of antenatal care. Qualitative findings revealed strong cultural influence on perceptions of birth defects, with causes attributed to medical factors (38.89%), supernatural beliefs such as witchcraft or curses (18.06%), immoral behaviour (12.50%), and dietary taboos (11.11%). Traditional health-seeking behaviour was common, with 91.67% consulting traditional healers during pregnancy. Linear regression analysis identified significant predictors of birth defects, including family history (β = 1.36, *p* = 0.008), alcohol use during pregnancy (β = 1.13, *p* = 0.050), and inadequate antenatal care attendance (β = 0.99, *p* = 0.040). Advanced maternal age showed a weaker and non-significant association (β = 0.79, *p* = 0.080). **Conclusions:** The study highlights substantial gaps in maternal knowledge and the strong influence of cultural beliefs on birth defect prevention. Strengthening culturally sensitive health education, improving antenatal care services, and engaging traditional healers in community-based interventions are essential to improve maternal health outcomes in rural South Africa.

## 1. Background

Congenital anomalies—also known as birth defects—are a significant global public health issue, affecting approximately 1 in every 33 live births. They account for over 300,000 infant deaths annually and often lead to long-term physical, cognitive, or developmental disabilities [1,2]. These abnormalities can be structural (such as neural tube defects or cleft palate) or functional (like metabolic or sensory disorders) and typically arise during early fetal development. Despite their profound impact, many of these conditions are preventable, particularly through cost-effective interventions such as folic acid supplementation, early antenatal care (ANC), and public health education [2,3].

In high-income regions like Europe, surveillance systems such as the EUROCAT network have improved epidemiological monitoring and resource allocation. However, challenges remain. The voluntary nature of folic acid supplementation before conception and the absence of mandatory food fortification in many countries have led to thousands of preventable cases of neural tube defects (NTDs) over recent decades [4]. North America, by contrast, has demonstrated the effectiveness of mandatory folic acid fortification, resulting in marked declines in NTD incidence and illustrating the value of policy-level interventions in reducing congenital anomalies [5].

Sub-Saharan Africa bears a disproportionate burden of congenital anomalies, with pooled prevalence rates reaching up to 23.5 per 1000 live births, and significantly higher in regions like Southern Africa [6]. Several factors contribute to this heightened risk: low maternal health literacy, poor access to prenatal vitamins, weak health infrastructure, and cultural practices that favor traditional over biomedical healthcare. Furthermore, deep-rooted beliefs in supernatural causes, such as witchcraft or curses, complicate efforts to promote scientific prevention and care strategies [7]. As a result, prevention campaigns have struggled to gain traction and remain largely ineffective without a concurrent cultural and systemic shift.

In South Africa specifically, congenital anomalies are among the leading causes of early neonatal mortality [8]. While the country has relatively well-developed health policies, implementation at the community level—especially in rural areas—remains a major obstacle. The Eastern Cape, one of the most rural and underserved provinces, illustrates these gaps vividly. Here, mothers face multiple barriers to effective access to ANC, including long distances to health facilities, limited transportation, financial constraints, and linguistic or cultural misunderstandings [9]. Compounding these issues is the widespread belief in traditional explanations for birth anomalies, which leads many women to seek care from traditional healers rather than formal medical services.

Addressing congenital anomalies in South Africa, and particularly in the Eastern Cape, requires a multi-level approach. This includes not only improving infrastructure and access to antenatal services but also engaging with communities through culturally sensitive education campaigns that dispel myths and promote biomedical explanations and prevention strategies. Integrating traditional health practitioners into formal health outreach initiatives may further enhance community trust and uptake of essential maternal health services [7,10].

Birth defects remain a major public health challenge globally, especially in low-resource rural settings like the Eastern Cape of South Africa. Despite the preventable nature of many congenital anomalies, maternal knowledge, attitudes, and practices (KAP) toward prevention are often inadequate and influenced by socio-cultural factors, including traditional beliefs and limited access to healthcare [6,9]. Understanding these multi-level contextual factors is essential for designing effective interventions tailored to local realities. Current data on maternal KAP regarding birth defect prevention in rural Eastern Cape is scarce, creating a critical gap that this study aims to fill. Women in rural districts of the Eastern Cape have limited knowledge and suboptimal attitudes and practices related to the prevention of birth defects, influenced by socio-cultural beliefs and inadequate access to antenatal care, which collectively increase the risk of birth defects in this population. Therefore, this study was conducted to assess maternal knowledge, attitudes, and practices regarding the prevention of birth defects and to explore the multi-level contextual factors influencing these in three rural districts of the Eastern Cape, South Africa.

## 2. Methods

### 2.1. Study Design

This study employed a cross-sectional mixed methods design to assess maternal knowledge, attitudes, and practices (KAP) regarding the prevention of birth defects in rural South Africa. The integration of quantitative and qualitative approaches allowed for both the measurement of KAP patterns and the exploration of contextual beliefs and behaviours influencing maternal health. This design is well-suited for public health research in underserved populations where cultural and structural factors strongly shape health outcomes [1].

### 2.2. Study Setting and Population

The study was conducted in three rural districts in the Eastern Cape Province, South Africa, purposively selected for their limited access to antenatal care (ANC), high maternal and neonatal risk profiles, and documented concerns regarding congenital anomalies. These settings are representative of rural South African communities characterised by socioeconomic constraints and strong traditional belief systems.

The study population comprised women aged 18–45 years who were either pregnant or had delivered within the past six months. A total of 72 women were included in the quantitative component. A formal sample size calculation was not conducted, as the study adopted a purposive sampling strategy to reach accessible, eligible participants within selected health facilities and community settings, rather than aiming for statistical generalisability.

For the qualitative component, participants were selected using maximum variation sampling to capture diverse experiences in age, parity, education, and socioeconomic status, ensuring depth and richness of contextual data.

### 2.3. Materials and Data Collection

Data were collected using two main instruments:

- Structured questionnaire (quantitative component): Administered in English only, this tool collected data on socio-demographic characteristics, obstetric history, knowledge of birth defects (e.g., folic acid use and causes), attitudes (e.g., cultural and spiritual beliefs), and practices (e.g., antenatal care attendance and substance use during pregnancy).
- Semi-structured interview guide (qualitative component): This explored maternal perceptions of congenital anomalies, reliance on traditional healers, pregnancy decision-making processes, and barriers to accessing formal healthcare. Interviews were conducted in isiXhosa or English based on participant preference, audio-recorded, transcribed verbatim, and translated where necessary.

### 2.4. Data Analysis

Quantitative data were analysed using IBM SPSS Version 27. Descriptive statistics (frequencies and percentages) were used to summarise participant characteristics and KAP variables.

Due to the relatively small sample size (N = 72) and the exploratory nature of the study, linear regression analysis was used instead of logistic regression to examine associations between maternal factors and birth defect outcomes. This approach was selected to avoid model instability and overfitting that may arise with small sample sizes in logistic models. Variables with *p* < 0.1 in univariate analysis were included in multivariable linear regression models. Regression coefficients (β), 95% confidence intervals, and *p*-values were reported to determine the strength and direction of associations.

Qualitative data were analysed using thematic analysis with NVivo software version 14. An inductive approach was applied, involving open coding, category development, and theme generation. This approach is appropriate for capturing culturally embedded beliefs, perceptions, and healthcare behaviours in rural and resource-limited settings [4].

### 2.5. Ethical Considerations

Ethical approval was obtained from the Walter Sisulu University Human Research Ethics Committee (Reference: WSU-REC/2025/08/06) [18 February 2019]. Informed consent was obtained from all participants after explaining the study’s purpose, procedures, and their rights, including voluntary participation and the option to withdraw at any time. All data were anonymized and stored securely to maintain participant confidentiality. The research adhered to ethical principles outlined in the Declaration of Helsinki.

## 3. Results

Most participants resided in the Amathole district (63.89%), followed by Alfred Nzo (18.06%) and Joe Gqabi (18.06%). Most women were aged between 20 and 35 years (52.78%), while 15.28% were younger than 20 years and 6.94% were older than 45 years. Over half of the respondents were single (55.56%), 34.72% were married, and the remainder were either separated (4.17%) or divorced (5.56%).

Most participants had primary education (56; 77.78%), followed by secondary (11; 15.28%) and tertiary education (5; 6.94%). The majority were unemployed (56; 77.78%), while smaller proportions were employed (10; 13.89%) or engaged in other income-generating activities (6; 8.33%), indicating limited participation in formal employment among respondents.

Nearly all participants (95.83%) reported having ever been pregnant. Among these, 36.11% had experienced 1–2 pregnancies, 30.56% had 3–4 pregnancies, and a small proportion (2.78%) had more than eight pregnancies. Pregnancy-related complications were commonly reported, with 70.83% of participants indicating that they had experienced one or more complications, while 26.39% had not, and 2.78% were unsure.

Most women (90.28%) delivered their most recent child at a health facility, whereas 9.72% delivered at home. Only 2.78% reported having had a child with a birth defect, while 97.22% reported no such history. However, 90.28% indicated a family history of birth defects.

Participants’ knowledge of birth defects varied. Genetic or inherited anomalies were the most recognized (69.23%). Traditional health-seeking practices were prevalent: 91.67% of women reported consulting traditional healers during pregnancy, reflecting the coexistence of traditional and biomedical belief systems.

Regarding perceptions of risk factors, although 93.06% acknowledged the harmful effects of alcohol consumption during pregnancy, only 18.06% identified smoking or medication use as potential risk factors, and 26.39% recognized advanced maternal age as a concern. Notably, only 13.89% believed that regular antenatal check-ups could help prevent birth defects. Table 1 summarizes the socio-demographic and reproductive characteristics of the study participants. Perceived causes of birth defects were diverse: 38.89% cited medical or biological factors, 18.06% attributed them to witchcraft or curses, and 12.50% to immoral lifestyles. Additional beliefs included dietary taboos (11.11%) and cultural rituals (4.17%), while 15.28% were unsure or reported multiple explanations.

### 3.1. Maternal Knowledge of Birth Defects

Most respondents demonstrated limited biomedical knowledge about birth defects. Although 77% were aware of folic acid’s role in prevention, only 31% understood the correct timing of supplementation (i.e., before conception or during the first trimester). Knowledge of behavioural and infectious risk factors, such as alcohol, tobacco, rubella, and diabetes, was low. Table 2 summarizes key findings on the knowledge of Risk Factors and Prevention.

### 3.2. Attitudes and Beliefs Toward Birth Defects

Traditional beliefs strongly influenced maternal attitudes. Nearly two-thirds (65%) attributed birth defects to supernatural causes, such as ancestral punishment or curses. Belief in spiritual healing was prevalent, and fatalistic views about pregnancy outcomes were common. Table 3 provides attitudes and Beliefs Toward Birth Defects. These findings point to deeply rooted cultural norms and beliefs impacting health-seeking behavior.

### 3.3. Maternal Practices and Predictors of Birth Defects

While 84% of women acknowledged the importance of ANC, only 62% attended ANC in the first trimester. Major barriers included fear of spiritual attack upon early pregnancy disclosure, transportation issues, fear of HIV testing, and poor patient–provider communication. Logistic regression showed that lack of early ANC, alcohol use during pregnancy, and family history of birth defects were significant predictors of birth defects. The linear regression analysis (Table 4) shows that family history of birth defects (β = 1.36, *p* = 0.008), alcohol use during pregnancy (β = 1.13, *p* = 0.050), and lack of regular antenatal care attendance (β = 0.99, *p* = 0.040) are significantly associated with increased risk of birth defects. Advanced maternal age showed a weaker and non-significant association in the adjusted model (β = 0.79, *p* = 0.080). Overall, the results (Table 4) indicate that both non-modifiable (family history) and modifiable factors (alcohol use and inadequate ANC) contribute to the risk of birth defects, highlighting key targets for maternal health interventions.

### 3.4. Qualitative Themes (In Vivo Representation of KAP Findings

Theme 1: “Folic acid helps, but many do not know when to take it.”

Participants generally recognised the importance of folic acid in preventing birth defects, with many acknowledging that it “prevents problems in pregnancy.” However, knowledge was incomplete and inconsistent, particularly regarding the timing of use. A dominant sub-theme reflected uncertainty, with many women indicating that they were “not sure when to start taking it,” suggesting gaps in antenatal counselling and early pregnancy health education.

Theme 2: “Bad habits like alcohol and smoking can harm the baby.”

Some participants demonstrated awareness of lifestyle-related risks, particularly alcohol and tobacco use during pregnancy. These were commonly described as behaviours that “can damage the baby.” However, this biomedical understanding coexisted with limited awareness of other medical or biological causes, indicating partial and fragmented risk knowledge.

Theme 3: “Illnesses and infections are not widely understood causes”

Very few participants associated congenital malformations with genetic or medical causes such as rubella infection or gestational diabetes. When mentioned, these were often described as “medical things I don’t really know about” or “hospital diseases,” reflecting low awareness of biomedical risk pathways and a reliance on general rather than specific knowledge.

Theme 4: “Some things are meant to happen or caused by ancestors.”

A strong cultural belief system emerged around the causes of birth defects. Many participants expressed that birth outcomes “are decided before birth” or that “ancestral spirits or curses can cause a child to be born with problems.” This fatalistic worldview shaped the understanding of pregnancy outcomes and often reduced perceived control over prevention.

Theme 5: “Spiritual healing is trusted alongside or above medical care”

Participants frequently expressed trust in traditional and spiritual healing practices. Many believed that “rituals or prayers can fix or prevent problems in pregnancy,” and some indicated greater confidence in traditional healers than in biomedical providers. This reflects a dual health belief system where spiritual interventions are seen as legitimate forms of care.

Theme 6: “Medical care helps, but is not always enough.”

While some participants acknowledged that medical services can “help manage pregnancy problems,” this view was not universal. A substantial proportion remained uncertain or sceptical about the effectiveness of biomedical care alone, often viewing outcomes as ultimately “in God’s or ancestors’ hands,” reinforcing a blend of medical and fatalistic beliefs.

### 3.5. Summary of Themes

Overall, the qualitative findings demonstrate a coexistence of partial biomedical knowledge and strong cultural–spiritual explanatory models. While awareness of folic acid and lifestyle risks exists, it is limited and inconsistent. More dominant are beliefs rooted in fatalism, ancestral influence, and spiritual causation, which strongly shape maternal attitudes and care-seeking behaviour.

## 4. Discussion

This study provides important insights into maternal knowledge, attitudes, and practices (KAP) regarding the prevention of birth defects among women in rural districts of the Eastern Cape, South Africa. The findings reflect a socioeconomically vulnerable population, with most participants residing in the Amathole district, being of reproductive age, having primary-level education, and experiencing high levels of unemployment. This profile is consistent with rural and under-resourced settings in South Africa, where limited education and economic constraints restrict access to health information and maternal healthcare services. High levels of pregnancy experience and reported complications further emphasise the need for strengthened maternal health interventions.

Despite relatively high awareness of certain risk factors, particularly alcohol use during pregnancy (93.06%), overall knowledge of birth defect prevention remained fragmented. While most participants were aware of folic acid as a preventive measure, only a minority understood the correct timing of intake, highlighting gaps in early pregnancy health education. Knowledge of other critical risk factors, including smoking, medication use, infections such as rubella, and gestational diabetes, was also limited. Although some participants recognised genetic causes, this did not translate into a comprehensive understanding of modifiable risks or prevention strategies. Notably, few participants identified antenatal care (ANC) as a preventive measure, despite high rates of facility-based delivery, indicating missed opportunities within the healthcare system to provide effective maternal health education.

The qualitative findings highlight the strong influence of cultural and spiritual belief systems on maternal perceptions and behaviours. Birth defects were commonly attributed to supernatural causes such as witchcraft, curses, and ancestral influence, alongside moral and dietary explanations. These beliefs were reflected in health-seeking behaviours, with many participants consulting traditional healers during pregnancy. The coexistence of biomedical and traditional explanatory models underscores the complexity of maternal health practices in this setting and aligns with existing evidence on cultural influences in rural African healthcare utilisation [11,12]. Fatalistic beliefs about pregnancy outcomes may further reduce perceived control over prevention and delay engagement with formal healthcare services.

These findings also reflect broader provincial and regional disparities in maternal health service utilisation. Similar to other rural provinces in South Africa, the Eastern Cape continues to experience delayed ANC initiation and gaps in preventive care despite national maternal health policies [13]. While interventions such as folic acid fortification have contributed to reductions in neural tube defects, inequalities in awareness and service access persist. Comparable patterns across sub-Saharan Africa show that limited maternal health literacy, socioeconomic barriers, and reliance on traditional medicine contribute to delayed care-seeking and suboptimal pregnancy outcomes [13,14,15].

The exploratory linear regression analysis identified family history of birth defects, alcohol use during pregnancy, and inadequate ANC attendance as factors associated with increased risk. These findings are consistent with existing evidence on both non-modifiable and modifiable determinants of congenital anomalies. However, these associations should be interpreted cautiously due to the small sample size and exploratory nature of the analysis. Logistic regression was not applied because key assumptions, including sufficient outcome events and model stability, were not met; therefore, linear regression was used to explore patterns of association while minimising the risk of overfitting and unstable estimates.

A key strength of this study is its mixed-methods design, which enabled the integration of quantitative findings with qualitative insights into culturally embedded beliefs and practices. This approach provides a more comprehensive understanding of maternal KAP in a rural context, where health behaviours are shaped by both structural and cultural factors [1,4]. The use of standardised and pre-tested KAP tools further enhances the reliability of the findings.

Several limitations should be acknowledged. The small sample size (N = 72) and purposive sampling limit the generalisability of the findings, and although stratification by age and education was used to reduce selection bias, the sample may not fully represent the broader population. The inclusion of participants from three districts reflects healthcare-seeking patterns and population mobility rather than strict geographic sampling. Additionally, reliance on self-reported data introduces potential recall and social desirability bias, and the cross-sectional design limits causal inference. As such, findings should be interpreted as exploratory and context-specific rather than definitive.

From a clinical and public health perspective, these findings highlight the need for culturally sensitive and context-specific maternal health interventions. Strengthening ANC services—particularly early initiation—is essential for improving maternal and neonatal outcomes. Health education should emphasise comprehensive risk communication, including appropriate folic acid use, avoidance of harmful substances, and early detection of complications. Importantly, interventions should engage with existing cultural belief systems, including collaboration with traditional healers and community structures, to improve acceptability and uptake [11,15].

At a policy level, strengthening maternal health education programmes, improving access to ANC services, and enhancing congenital anomaly surveillance systems are critical. Community-based strategies, including the involvement of community health workers, may help bridge gaps between formal healthcare systems and rural populations. Future research should focus on larger, more representative samples and longitudinal or interventional designs to better understand causal pathways and evaluate the effectiveness of culturally tailored interventions.

## 5. Conclusions

Maternal knowledge, attitudes, and practices in the Eastern Cape remain strongly influenced by cultural norms, with limited biomedical understanding and inconsistent engagement with antenatal care services. Comparisons across South Africa, the African region, and global contexts highlight persistent inequalities in access to care, health literacy, and implementation of preventive strategies. Addressing these gaps requires coordinated, multi-level interventions that integrate culturally appropriate health education, strengthened healthcare systems, and evidence-based policy implementation. Future efforts should prioritize community engagement, integration of traditional and formal healthcare systems, and equitable access to antenatal care to reduce the burden of preventable birth defects.

## Figures and Tables

**Table 1:** Summarizes the socio-demographic and reproductive characteristics of the study participants.

Variable	Category	Frequency	Percent	Cumulative %
1. District	Alfred Ndzo	13	18.06%	18.06%
	Joe Gqabi	13	18.06%	36.11%
	Amathole	46	63.89%	100.00%
	Total	72	100%	
2. Age	<20 years	11	15.28%	15.28%
	20–35 years	38	52.78%	68.06%
	36–45 years	18	25.00%	93.06%
	>45 years	5	6.94%	100.00%
	Total	72	100%	
3. Marital Status	Single	40	55.56%	55.56%
	Married	25	34.72%	90.28%
	Separated	3	4.17%	94.44%
	Divorced	4	5.56%	100.00%
	Total	72	100%	
4. Level of Education Attained	Primary	56	77.78%	77.78%
	Secondary	11	15.28%	93.06%
	Tertiary	5	6.94%	100.00%
	Total	72	100%	
5. Source of Income	Unemployed	56	77.78%	77.78%
	employed	10	13.89%	91.67%
	Others	6	8.33%	100.00%
	Total	72	100%	
6. Religious Affiliation	Christian	70	97.22%	97.22%
	Others	2	2.78%	100.00%
	Total	72	100%	
7. Have You Ever Been Pregnant?	Yes	69	95.83%	95.83%
	No	3	4.17%	100.00%
	Total	72	100%	
8. Number of Pregnancies	1–2	26	36.11%	36.11%
	3–4	22	30.56%	66.67%
	5–6	14	19.44%	86.11%
	7–8	8	11.11%	97.22%
	>8	2	2.78%	100.00%
	Total	72	100%	
9. Experienced Any Pregnancy Event(s)?	Yes	51	70.83%	70.83%
	No	19	26.39%	97.22%
	Not sure	2	2.78%	100.00%
	Total	72	100%	
10. Place of Last Delivery	Health facility	65	90.28%	90.28%
	Home	7	9.72%	100.00%
	Total	72	100%	
11. Had a Baby with a Birth Defect?	No	70	97.22%	97.22%
	Yes	2	2.78%	100.00%
	Total	72	100%	
12. Family History of Birth Defects?	Yes	65	90.28%	90.28%
	No	7	9.72%	100.00%
	Total	72	100%	

**Table 2:** Knowledge of Risk Factors and Prevention (N = 72).

Knowledge Item	Yes (%)	No (%)
Aware that folic acid prevents birth defects	77.0	23.0
Knows the correct timing of folic acid intake	31.0	69.0
Alcohol and tobacco use are risk factors	58.0	42.0
Aware of genetic/inherited causes	18.0	82.0
Aware of rubella infection as a risk factor	16.0	84.0
Recognizes gestational diabetes as a risk factor	19.0	81.0

**Table 3:** Attitudes and Cultural Beliefs (N = 72).

Belief/Attitude	Agree (%)	Disagree/Neutral (%)
Birth defects are caused by supernatural forces (e.g., curses)	65.0	35.0
Spiritual or ritual healing can cure or prevent birth defects	71.0	29.0
Birth outcomes are predetermined/fated (“meant to be”)	63.0	37.0
Trust in traditional healers over biomedical health providers	59.0	41.0
Birth defects can be managed with medical care	47.0	53.0

**Table 4:** Linear Regression Analysis of Factors Associated with Birth Defects (N = 72).

Factors	Unstandardized Coefficient (β)	*p*-Value	Adjusted β	*p*-Value
Family history of birth defects	1.53	0.001	1.36	0.008
Alcohol use during pregnancy	1.34	0.020	1.13	0.050
No regular ANC attendance	1.10	0.015	0.99	0.040
Advanced maternal age (≥35 years)	0.92	0.030	0.79	0.080

## Data Availability

The data presented in this study are available on request from the corresponding author.

## Abbreviations

ANC	Antenatal Care
KAP	Knowledge, Attitudes, and Practices
NTDs	Neural Tube Defects
CHWs	Community Health Workers
LMICs	Low- and Middle-Income Countries
